# An Attention Model With Transfer Embeddings to Classify Pneumonia-Related Bilingual Imaging Reports: Algorithm Development and Validation

**DOI:** 10.2196/24803

**Published:** 2021-05-17

**Authors:** Hyung Park, Min Song, Eun Byul Lee, Bo Kyung Seo, Chang Min Choi

**Affiliations:** 1 Department of Pulmonary and Critical Care Medicine Asan Medical Center Seoul Republic of Korea; 2 Yonsei University Seoul Republic of Korea; 3 Department of Oncology Asan Medical Center Seoul Republic of Korea

**Keywords:** deep learning, natural language process, attention, clinical data, pneumonia, classification, medical imaging, electronic health record, machine learning, model

## Abstract

**Background:**

In the analysis of electronic health records, proper labeling of outcomes is mandatory. To obtain proper information from radiologic reports, several studies were conducted to classify radiologic reports using deep learning. However, the classification of pneumonia in bilingual radiologic reports has not been conducted previously.

**Objective:**

The aim of this research was to classify radiologic reports into pneumonia or no pneumonia using a deep learning method.

**Methods:**

A data set of radiology reports for chest computed tomography and chest x-rays of surgical patients from January 2008 to January 2018 in the Asan Medical Center in Korea was retrospectively analyzed. The classification performance of our long short-term memory (LSTM)–Attention model was compared with various deep learning and machine learning methods. The area under the receiver operating characteristic curve (AUROC), area under the precision-recall curve, sensitivity, specificity, accuracy, and F1 score for the models were compared.

**Results:**

A total of 5450 radiologic reports were included that contained at least one pneumonia-related word. In the test set (n=1090), our proposed model showed 91.01% (992/1090) accuracy (AUROCs for negative, positive, and obscure were 0.98, 0.97, and 0.90, respectively). The top 3 performances of the models were based on FastText or LSTM. The convolutional neural network–based model showed a lower accuracy 73.03% (796/1090) than the other 2 algorithms. The classification of negative results had an F1 score of 0.96, whereas the classification of positive and uncertain results showed a lower performance (positive F1 score 0.83; uncertain F1 score 0.62). In the extra-validation set, our model showed 80.0% (642/803) accuracy (AUROCs for negative, positive, and obscure were 0.92, 0.96, and 0.84, respectively).

**Conclusions:**

Our method showed excellent performance in classifying pneumonia in bilingual radiologic reports. The method could enrich the research on pneumonia by obtaining exact outcomes from electronic health data.

## Introduction

Electronic health records (EHRs) have become increasingly incorporated into clinical practices in hospitals over the past few decades [1]. EHR data are voluminous and can be used as real-world evidence if they are analyzed with proper methods [2]. However, the data are not collected for research purposes [2], and several rule-based methods are used to extract particular outcomes from the data set. There have been numerous studies where analyses were performed using EHR data with labels such as *sepsis* defined by rule-based outcomes [3-6]. However, defining outcomes other than laboratory findings is difficult because the data are unstructured and written as natural language. For this reason, a previous study that used the outcome *pneumonia* defined pneumonia by its International Classification of Diseases, Ninth Revision, Clinical Modification (ICD-9-CM) code [7,8]. However, the use of ICD codes as a label does not contain temporal information, such as the exact time of diagnosis during hospital admission, and it is hard to perform time series analysis with this limited information.

Although medical imaging reports contain a great deal of information regarding diagnosis and clinical features, it is hard to analyze the information because they are formatted as unstructured free text and are variably written depending on the radiologist.[9] For this reason, medical imaging reports are rarely used as outcomes in big data analysis [10]. However, as long as pneumonia can be identified in radiologic reports, other important information, such as the time of onset and the presence of pneumonia during admission, can also be derived. Moreover, labeled data are essential in deep learning because the analysis requires millions of observations to reach acceptable performance levels [11].

As of 2018, 43 studies using natural language processing for the identification of chronic diseases in EHRs had been published, and only recently have there been more studies conducted on this topic using deep learning [12]. Especially in deep learning, convolutional neural network (CNN)–based models have shown significant accuracy in extracting pulmonary embolism [10] and pulmonary infection from medical reports [1]. The model can be used to classify diagnosis from whole medical records even when they are written in the Chinese language [13], and a recurrent neural network–based model has been used for classifying stroke and identifying its location [14]. However, the use of bilingual clinical reports is common for EHRs in non–English-speaking countries.

The purpose of our study was to classify reports of pneumonia consisting of findings derived during the pre- and postoperative period of a major surgery that were written as bilingual texts (English and Korean). We compared the performance of traditional models with deep learning models, with the latter showing excellent performance in previous studies, and identified the best performing model as an attention-based bidirectional long short-term memory (Bi-LSTM) model neural network.

## Methods

### Clinical Data

We retrospectively included radiology reports for chest computed tomography (CT) and chest x-rays of surgical patients from January 2008 to January 2018 in the Asan Medical Center in Korea. The patients had undergone upper abdominal and thoracic surgeries, as coded by the ICD-9-CM. Detailed criteria for the surgery are described in Multimedia Appendix 1.

The radiology reports consist of chest CT and chest x-rays (posteroanterior and anteroposterior) that are extracted by radiology procedure codes. The chest x-ray reports have no structured format and only contain descriptions. The chest CT reports consist of the short history of the patients, the findings, and a conclusion; however, the format varies depending on the writing style of the radiologist. The conclusions in around half of the chest CT reports were omitted due to the different writing style of the radiologists. Therefore, we used only the findings of chest CT and the descriptions of chest x-rays to classify the labels, and all the annotation was based solely on the description of each report.

Usually, the pneumonia incidence in surgical patients is around 1%, suggesting that reports of pneumonia are rare. To overcome the imbalance of the positive and negative data sets, we only included radiologic reports that contained pneumonia-related words. The words representing pneumonia were as follows: “pneumoni-,” “consolid-,” “infiltra-,” “bronchiole-,” “hazi-,” “hazzi-,” “opacit-,” and “GGO”.

From a total of 1,088,680 radiology reports, 886,248 were included after reports with inappropriate surgical procedures were excluded. The detailed inclusion criteria of the appropriate procedures have been described in a previous study [3]. After extracting the pneumonia-related words, 23,377 reports were included.

### Report Annotation

**Figure 1:**
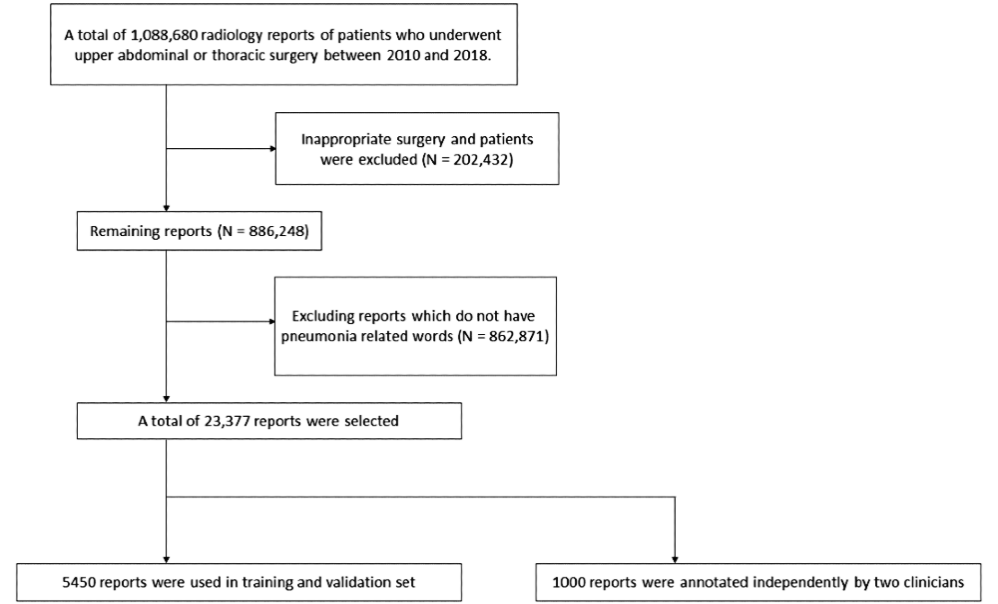
Radiologic reports flowchart.

Among the 23,377 reports, a total of 5450 annotated reports were used to train our model. A clinician annotated the 5450 reports and used them for training and validation. After training the model, 2 different clinicians, who worked independently from the first clinician, annotated another 1000 reports for an extra-validation set (Figure 1).

All document-level annotations by clinicians included 3 categories for pneumonia: negative, positive, and unclear (obscure). The positive pneumonia reports included postoperative infection reports and did not contain reports for noninfectious diseases, such as organizing pneumonia or interstitial lung disease, because the label was required to represent pneumonia as a perioperative complication. The excluded reports were labeled as negative reports. It was observed that 895 reports were pneumonia positive, 4005 reports were pneumonia negative, and 550 reports were obscure results. In the extra-validation set, 2 clinicians independently labeled the radiologic reports on the basis of the clinical importance of the findings. To overcome the human error of the 2 clinicians, the consensus label of the 2 clinicians was regarded as the reference standard. An interrater reliability (k score) was calculated by Cohen κ value.

### Ethics Approval

This study was approved by the ethics committee of the Asan Medical Center (approval no. 2018-1122), and the need to obtain informed consent was waived because of the retrospective observational nature of the study. The clinical data that were extracted using the Asan Biomedical Research Environment system were indexed by deidentified encrypted patient ID numbers so that the researchers would not be able identify the patients [15,16].

### Proposed Approach

As most of the verbs and adjectives in clinical reports are written in Korean, and most of nouns (usually the names of the diseases) are written in English, we had to consider 2 different languages. Therefore, we proposed a new method for a bilingual clinical data set based on the classification algorithm of combining substring and translation embeddings (Kor2Eng) with an attention-based Bi-LSTM neural network (LSTM-Attention). Multimedia Appendix 1 Figure S4 shows the architecture of our proposed model.

The proposed method includes 3 steps: (1) text preprocessing; (2) word representation, which is composed of substring and Korean-to-English (Kor2Eng) embeddings; and (3) training of the classification model.

Our data set, which is a description of x-ray and CT, is composed of a mix of Korean and English sentences. Therefore, specific preprocessing is required before the statements are fed into the classification model. The detailed methods for text preprocessing and training are described in Multimedia Appendix 1.

### Kor2Eng Transfer Embedding

Training word vectors require a considerable amount of data and time. Therefore, we applied embeddings by training them independently on monolingual data and pretraining them with Wikipedia data. However, due to the characteristics of data, the text of the clinical notes was a mixture of English and Korean. If a monolingual embedding were to be used for this data, one side of the information would be lost. To reduce the loss of information, we used a translation method that converts the vector of Korean words into the vector of English words with similar meanings. The unsupervised method of translating the source language into the target language was proposed by Lample et al [17]. In this method, the process of learning a mapping occurs between the 2 sets of embedding in the shared space. We trained the subword embedding model to learn Korean-to-English mapping using the unsupervised method without any parallel data.

### Deep Learning–Based Classification Model

We built an attention-based deep neural network using LSTM. LSTM is a recurrent neural network variant that alleviates the vanishing gradient problem by learning and remembering long-term dependencies [18] and consists of a cell memory state and 3 gates.

The Bi-LSTM consists of a forward–backward LSTM layer [19]. Both layers are connected to the same output layer. Our classification model used Bi-LSTM with the attention mechanism. This allowed the model to simultaneously handle information from different positions.

**Figure 2:**
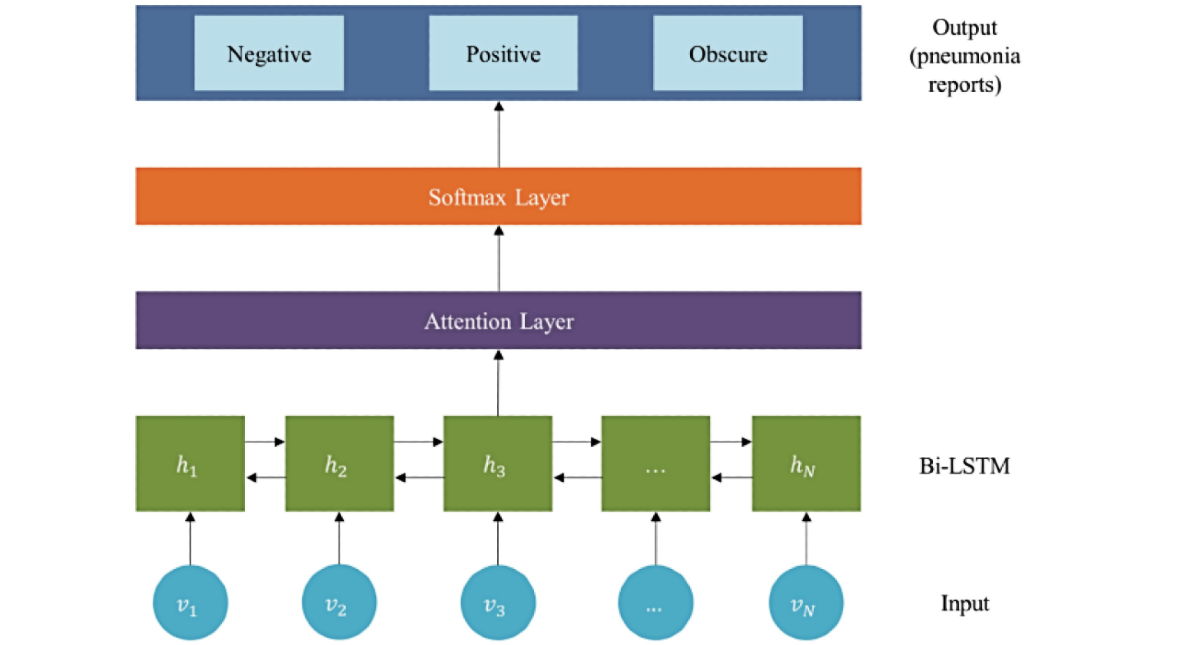
The architectures of a deep learning-based classification model. Each input receives an embedding of English translated from Korean. In the attention layer, each word has an attention weight which is translated into the importance for prediction. Bi-LSTM: bidirectional long short-term memory model.

Figure 2 shows the architecture of the deep learning–based classification model. First, the input is fed into the Bi-LSTM layer. Second, the output of the Bi-LSTM layer is fed into the attention layer (Bi-LSTM–Attention) for attending important words. Finally, the output of the attention weight passes through the softmax layer for classification.

The performance metrics (ie, precision, recall [sensitivity], and *F*_1_ score) were used to evaluate the models. The accuracy, area under the receiver operating characteristic curve (AUROC), and area under the precision-recall curve (AUPRC) were used to compare the models. For analyzing the multilabel data set, labels were treated as interested labels and other labels in evaluating each metric. For example, when we treated the precision for negative labels, only the true negative data were treated as true labels while positive and obscure labels were treated as false labels. *F*_1_ score is the weighted average of precision and recall, and it is used to measure the performance of a model when the data consist of uneven class distributions [20]. The statistical analysis was performed on Python 3.7.6 (Python Software Foundation).

## Results

In this section, we evaluated the performance of the various classification models. To demonstrate the performance of our method, we compare the proposed model with traditional machine learning and other deep learning models. The machine learning models included logistic regression [21], support vector machine [22], Naïve Bayes regression [23], K-nearest neighbors algorithm [24], decision tree [25], and random forest [26]. The deep learning models included the word-to-vector representation model (Word2Vec) [27], FastText [17], CNN [28], and LSTM [29]. The details of each model are described in Multimedia Appendix 1.

Out of 5450 data sets, 4005 did not contain pneumonia, 895 contained pneumonia, and 550 were obscure, with 80% being used in the training set and the remaining 20% in test set. The test set was composed of no pneumonia (n=801), pneumonia (n=179), and obscure (n=110) classifications. The extra-validation set was annotated by 2 independent clinicians. Out of a total of 1000 radiologic reports, 803 labels were agreed upon by 2 independent clinicians. Among these labels, 498 did not contain pneumonia, 185 contained pneumonia, and 120 were obscure cases.

### Accuracy of Our Model as Compared to Previous Models

We evaluated the performance of the different models to find the best model. As shown in Table 1, the prediction accuracy changed depending on the model. The traditional models (ie, support vector machine, Naïve Bayes, etc) achieved an accuracy between 64.03% and 83.03%. The logistic regression showed a reasonable performance with an accuracy of 83.03% (Multimedia Appendix 1 Table S1).

The deep learning–based methods (ie, FastText, Word2Vec with Bi-LSTM–Attention, and the proposed model) outperformed the traditional models. The prediction accuracy of the deep learning models was 90.00%, 88.99%, and 91.01% for FastText, Word2Vec with Bi-LSTMAttention, and the proposed model, respectively. These deep learning models showed a 10% higher accuracy than did the traditional machine learning methods because sentence classification required the interpretation of complex features. The proposed model achieved the highest performance compared to the other deep learning models (Multimedia Appendix 1 Table S1).

### Model Accuracy Based on the Different Representation Methods of Words

We evaluated the performance based on different methods of word representation. The Word2Vec with Bi-LSTM–Attention model is a more commonly used language representation model. The model showed a higher accuracy and *F*_1_ score than did the traditional models; however, the drawback associated with this model is that the foreign language is not represented (Table 1). We implemented another representation method with a substring using the FastText model. This method involves slicing of words to bunches of characters, which can be a better expression for the foreign language. The substring with FastText model achieved a precision of 93% for negative, 84% for positive, and 74% for obscure classifications; and a recall of 93% for negative, 84% for positive, and 47% for obscure classifications. The substring with FastText model showed a better performance than did the Word2Vec model according to *F*_1_ score.

Our proposed model (Kor2Eng) translated Korean to English before the prediction process. The proposed model achieved a precision of 96%, 86%, and 61%, and a recall of 97%, 80%, and 64% for positive, negative, and obscure classifications, respectively. The AUROC of the model was 0.98 for negative, 0.97 for positive, and 0.90 for obscure classifications, while the AUPRC was 0.99 for negative, 0.87 for positive, and 0.62 for obscure classifications (Multimedia Appendix 1 Figure S5). Compared to the classification of the negative labels, which was a relatively easy task (96% of negative), classifying positive or obscure labels was a harder task and showed a rather lower *F*_1_ score (83% for positive and 62% for obscure). For classifying the obscure classification, our model showed the highest performance among different representation methods (substring with FastText, Word2Vec, and Kor2Eng).

**Table 1:** The detailed performance of the top 3 best-performing models.

Models			Precision, n/N (%)		Recall, n/N (%)			*F*_1_ score (%)			AUROC^a^		AUPRC^b^
**Substring+FastText** [17]													
	Negative	776/819 (94.7)				776/801 (96.9)			96		0.82		0.92
	Positive	153/593 (25.8)				153/179 (85.5)			83		0.74		0.34
	Obscure	52/73 (71.2)				52/110 (47.3)			57		0.71		0.22
**Word2Vec^c^+Bi-LSTM^d^–Attention**													
	Negative	772/849 (90.9)		772/801 (96.4)			94			0.95		0.98	
	Positive	153/222 (68.9)		153/179 (85.5)			81			0.96		0.87	
	Obscure	47/80 (58.8)		47/110 (42.7)			49			0.88		0.51	
**Proposed model (Kor2Eng^e^)**													
	Negative	776/809 (95.9)		776/801 (96.9)			96			0.98		0.99	
	Positive	153/182 (84.1)		153/179 (85.5)			83			0.97		0.87	
	Obscure	70/115 (60.9)		70/110 (63.6)			62			0.90		0.62	

^a^AUROC: area under the receiver operating characteristic curve.

^b^AUPRC: area under the precision-recall curve.

^c^Word2Vec: the word-to-vector representation model.

^d^Bi-LSTM: bidirectional long short-term memory model.

^e^Kor2Eng: Korean to English.

### Visualization of Relative Importance

**Figure 3:**
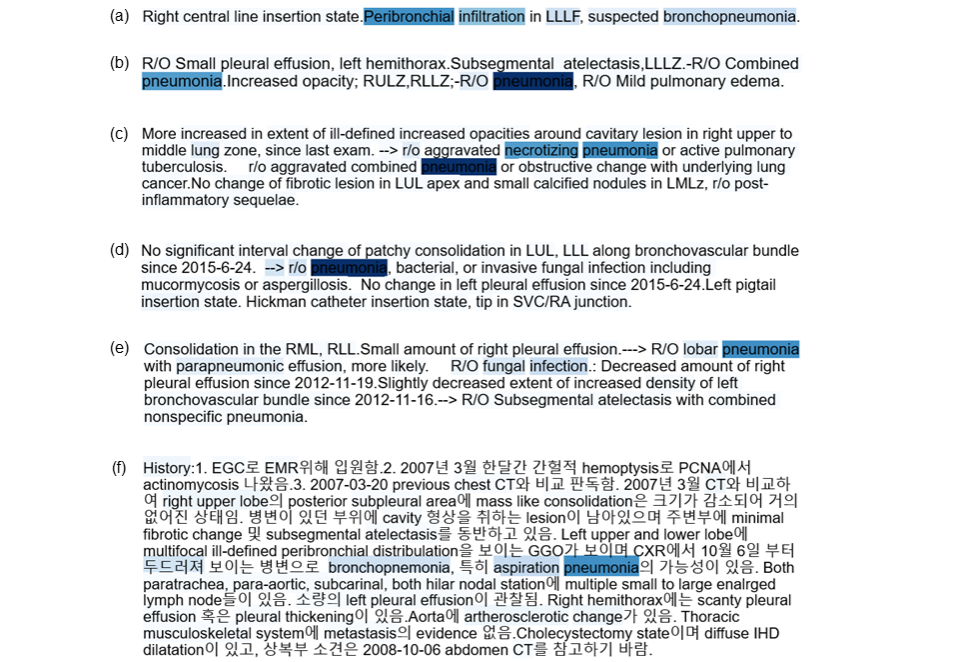
Visualization of the importance of words by attention weights.The darker the color is, the greater the importance of the words for predicting the pneumonia label. High attention weight is depicted in the darker color. Words with high attention weights are shown.

We visualized the weighted words when the proposed model classified the input data. In the attention model, the weight of each word could be used for classifying the reports. Based on the intensity of color, the importance of a word was indicated when the proposed model determined the class of the input data. Darker colors indicated a higher importance for classifying pneumonia. Figure 3 shows the instances where the proposed model predicted pneumonia reports correctly. For example, the highlighted words “Peribronchial,” “infiltration,” “suspected,” and “bronchopneumonia” indicate pneumonia (Figure 3a). In the bilingual texts (Figure 3f), the following words are important to classifying pneumonia-reports: “두드러져,” “bronchopneumonia,” “aspiration,” and “pneumonia.”

### Extra Validations

**Figure 4:**
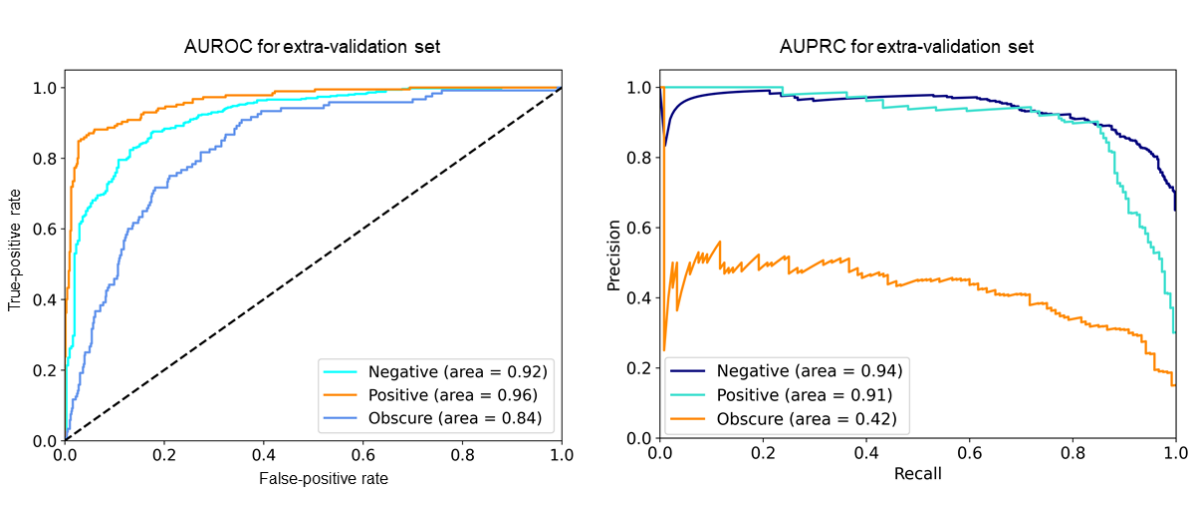
AUROC and AUPRC of our proposed model in the extra-validation set. AUROC: area under the receiver operating characteristic curve; AUPRC: area under the precision-recall curve.

As an extra validation of our proposed model, 2 clinicians labeled an additional data set. The data set was randomly selected from the entire data set, excluding the previously trained data. For precise labeling, 2 medical doctors each labeled the records. Of the 1000 records, 803 were agreed upon by 2 independent physicians. The Cohen κ value of the clinicians’ label was 0.63 (95% CI 0.59-0.67). Table 2 shows the performance results of the proposed model with the extra-validation data set. The AUROC and AUPRC for positive labels were slightly lower in the extra-validation set than in the test set (Figure 4). The *F*_1_ score of positive labels was similar to that of the training data; however, predicting negative and obscure labels showed a relatively poor performance as compared to the training data set according to *F*_1_ score. The overall accuracy of our model was 80.0%.

**Table 2:** Extra validation of the proposed Korean-to-English (Kor2Eng) model.

Class	Precision, n/N (%)	Recall, n/N (%)	*F*_1_ score	AUROC^a^	AUPRC^b^
Negative	422/470 (89.8%)	422/498 (84.7%)	87%	0.92	0.94
Positive	142/155 (91.6%)	142/185 (76.8%)	84%	0.96	0.91
Obscure	77/178 (43.3%)	77/120 (64.2%)	52%	0.84	0.42

^a^AUROC: area under the receiver operating characteristic curve.

^b^AUPRC: area under the precision-recall curve

## Discussion

The purpose of the Kor2Eng model is to classify pneumonia-related medical records written in Korean and English. Our proposed model showed 91.01% accuracy in the test set and 80.0% accuracy in the extra-validation set for classifying pneumonia reports. Appropriate classification of radiologic reports is mandatory for further analysis regarding pneumonia through EMRs. As compared to other models, such as CNN or traditional machine learning models, our model showed better performance. The 3 best-performing models (Word2Vec with Bi-LSTM–Attention, FastText, and the proposed model) demonstrated better performance than did the traditional and CNN models, and our proposed model provided the highest AUROC and AUPRC among the top 3 models. Because too many false-positives may lead to clinician exhaustion, a model with excellent performance is desirable. We consider that a model with an AUROC of at least 0.95 can be used in clinical practice or for labeling the data set. The false-positive results of pneumonia reports can be additionally filtered with other clinical findings such as respiratory symptoms or antibiotics use, as pneumonia is defined by respiratory symptoms with radiologic findings [30].

The label balance of the data set was a consequence of excluding irrelevant labels to our target. As the reports that do not have pneumonia-related words can be considered pneumonia-negative radiologic reports, the reports requiring classification must contain at least one of the pneumonia-related words such as “consolidation” or “haziness”. Excluding the irrelevant label is clinically appropriate and balances the data set with each label, with the balanced data set mitigating the overestimation of the model. Furthermore, filtering radiologic reports containing relevant words might make the data set rather homogenous, which makes classification a hard task. Our model showed an excellent performance in classifying pneumonia, and thus, it can be used for auto-labeling in classifying pneumonia reports.

A notable observation is the discrepancy between the test and extra-validation set. The model showed a rather similar performance in classifying negative and positive cases and a relatively poor performance in obscure cases. One reason for this discrepancy might be that 2 different clinicians annotated the entire extra-validation set. As some of the obscure cases are classified by the nuance of the context, the 2 clinicians might have differed in labeling the obscure cases. Therefore, the labeling of the obscure classification in the extra-validation set might have been different from that of the training set. The pneumonia cases in the report should only be decided by clinical situations, and thus, the importance of obscure cases should be evaluated in subsequent studies.

Several studies have been conducted for classifying radiologic reports as positive or negative for a given disease [1,10,31,32] or for classifying various diagnoses from medical records written in Chinese [13]. Most of the studies used a CNN-based model and showed a better performance than did our model [1,10,31,32]. In our study, we compared several deep learning models from logistic regression to LSTM with attention. The CNN model, which showed an excellent performance in previous studies [1,10,31,32], was inferior to the attention-based LSTM model in our data set. The reason for its relatively poor performance might be explained by our data selection. We selected radiologic reports that had at least one of the pneumonia-related words. This selection made the radiologic reports relatively homogeneous compared to those used in previous studies, which might contain a wider variety of radiologic reports. As we compared the performance with the CNN model, our proposed model was found to be comparably accurate with those of previous studies and showed better performance.

Radiologic reports in this study consisted of 2 languages: English and Korean. Compared to the English data set, the Korean word data set has a lack of studies in embedding and analyzing in deep learning. To overcome this limitation, we used unsupervised translation of Korean words to English words, which had pretrained embedding [17]. Compared to the Word2Vec with Bi-LSTM–Attention model, the attention/LSTM model with transfer embedding showed a better performance in classification, especially for obscure labels. This method might be especially important in bilingual reports.

Our study has several limitations. First, we only included reports from a single tertiary center of surgical in-patients. Our model might be inaccurate in a reporting style different from the one that we have incorporated. Thus, if the model used a data set from another reporting style, the model would need to be validated again. However, in this case, more labeled data might be available, and thus the applied method would show better performance in another data set, especially for bilingual text reports. Second, we could not compare the exact same models with the previous models that showed good performance. However, we compared our model with various deep learning models that were used in previous studies, which is sufficient to compare the performance of different model structures.

In summary, our proposed model showed superior performance as compared to other algorithms in the classification of pneumonia from radiologic reports. In bilingual radiologic reports, the proposed method of transferring and Bi-LSTM–Attention model showed significant improvement in performance than did the previous high-performing models. We hope that this method could be used to enrich the research about pneumonia by obtaining exact outcomes from electronic health data.

## Appendix

Multimedia Appendix 1 Supplementary method and figures.

## Abbreviations

AUPRC: area under the precision-recall curve
AUROC: area under the receiver operating characteristic curve
Bi-LSTM: bidirectional long short-term memory model
CNN: convolutional neural network
CT: computed tomography
EHR: electronic health record
ICD-9-CM: International Classification of Diseases, Ninth Revision, Clinical Modification
Kor2Eng: Korean to English
LSTM: long short-term memory model
Word2Vec: word-to-vector representation model
