# Supplementary material for: An Attention Model With Transfer Embeddings to Classify Pneumonia-Related Bilingual Imaging Reports: Algorithm Development and Validation
Source: JMIR Med Inform. 2021 May 17;9(5):e24803. doi: 10.2196/24803 (PMC8167619; doi:10.2196/24803)
Supplement: Multimedia Appendix 1 [file medinform_v9i5e24803_app1.docx]

Supplement

Classifying Pneumonia-Related Bilingual Imaging Reports (Attention Model With Transfer Embeddings): Algorithm Development and Validation

*Hyung Jun Park^1^, Min Song^2^, Bo Kyung Seo^2^, Eun Byul Lee^2^, and Chang Min Choi^1,3^*

**Affiliations**: ^1^Department of Pulmonary and Critical Care Medicine, Asan Medical Center, University of Ulsan College of Medicine, Seoul, South Korea ^2^Affiliation, Department of Digital Analytics, Yonsei University ^3^Affiliation, Department of Oncology, Asan Medical Center, University of Ulsan College of Medicine, Seoul, South Korea

**Corresponding authors:** Chang-Min Choi

**Corresponding author contact details:** Department of Pulmonary and Critical Care Medicine and Department of Oncology, Asan Medical Center, University of Ulsan College of Medicine, 88, Olympic-ro 43-gil, Songpa-gu, Seoul 05505, South Korea ccm9607@gmail.com

Phone: +82-2-3010-5902

Fax: +82 02-3010-6968

**Sources of support:** This work was supported by the Bio-Synergy Research Project (NRF-2013M3A9C4078138) of the Ministry of Science, ICT, and Future Planning through the National Research Foundation.

**Running head:** Deep learning for pneumonia-text classification

Table of Contents

[Detailed method of the proposed model 3](#_Toc71471419)

[Text Preprocessing 3](#_Toc71471420)

[Appendix Figure S1. The entire process of text preprocessing 3](#_Toc71471421)

[Appendix Figure S2. The example of text preprocessing processes 4](#_Toc71471422)

[Word Representation 4](#_Toc71471423)

[Appendix Figure S3. The architecture of substring embedding model 5](#_Toc71471424)

[Structure of our Deep-learning based classification model 6](#_Toc71471425)

[Bi-LSTM Layer 6](#_Toc71471426)

[Attention Layer 6](#_Toc71471427)

[Softmax Layer 7](#_Toc71471428)

[Training hyper-parameters in our proposed model 7](#_Toc71471429)

[Other models compared to our proposed model 8](#_Toc71471430)

[Appendix Table S1. Comparison of traditional and deep learning models 10](#_Toc71471431)

[Appendix Figure S4. Overall processing of our proposed model 11](#_Toc71471432)

[Appendix Figure S5. AUROC and AUPRC of our proposed model in extra-validation set. 12](#_Toc71471433)

[Reference 13](#_Toc71471434)

## Detailed method of the proposed model

## Text Preprocessing

As shown in Appendix Figure S1 which overviews the entire process of text pre-processing, we applied a special technique that is different from conventional pre-processing in the present study because the statements were written in both Korean and English.

### Appendix Figure S1. The entire process of text preprocessing


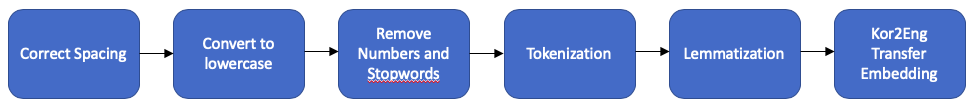


Appendix Figure S2 shows how the preprocessing was applied on an original text. First, the whole text is converted to lowercase to put all words on same level. Second, the numbers, stop-word, and special characters are removed from the original text. In the statements, there are spacing errors, such as extra spaces after a word with the quotation mark. We then applied tokenization and lemmatization to the text. Lemmatization is to find the base form of a word used in various forms in sentences. It aims to reduce inflectional forms. In addition, we eliminated word where English is followed by Korean postpositions which are suffixes or short words in Korean grammar that immediately follow a noun or pronoun. Appendix Figure S2-(d) shows the task before the removal of Korean postpositions in text, and Supplement Figure 2-(e) shows text containing red words which are eliminated Korean postpositions in text.

The n-gram is the use of consecutive n words as a single word. A token unit consisting of two consecutive words is called bigram. The unigram is commonly used to represent a document as a bag-of-words model. However, when it comes to document classification, the bigram has better information power than the unigram (1). Supplement Figure 2-(f) shows bigram of words.

### Appendix Figure S2. The example of text preprocessing processes


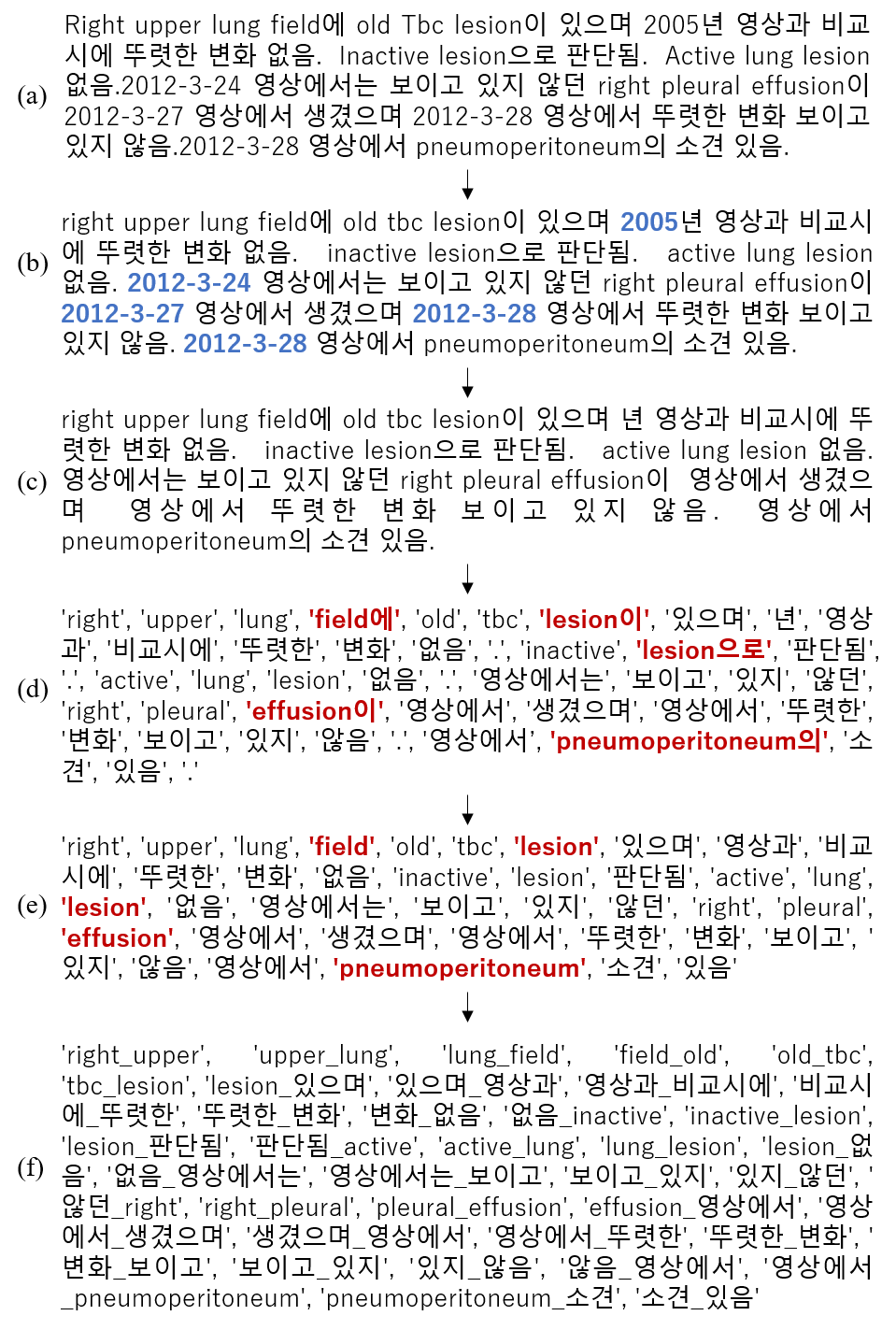


(a) original text (b) convert to lowercase (c) remove numbers (d) tokenization and lemmatization (e) remove Korean postpositions after English (f) bigram

## Word Representation

One-hot encoding is the easiest way to change words to vectors. This is how each N word is expressed as an N-dimensional vectors, which puts 1 in the place where the word is included and 0 in the rest. Thus, one-hot vectors take the form of sparse vectors, with only one element 1 and the rest all zero. This means that they exist independently without any specific relationship with each other or meaning, such as synonym or antonym.

The previous language model has several limitations. The parameter is inaccurate for words with low frequency as well as out-of-vocabulary (OOV) words because it requires large training data to optimize the parameter. Also, by encoding with a one-hot vector, information inside the word structure cannot be utilized. To deal with these problems, we applied the sub-word level embedding method (2).

In this method, words are regarded as a bag-of-characters, and characters of n-grams are embedded rather than individual words. We assume that given a dictionary of sub-words of size S, the set of sub-word appears in given word $w$ as $\mathcal{S}_{w}\subset\{1, 2, \ldots, S\}$. As shown in Supplement Figure S3, $w$ has several sub-words $\mathcal{S}_{w}^{1}, \mathcal{S}_{w}^{2}, \ldots,\mathcal{S}_{w}^{s}$ and each sub-word is embedded according to the Skip-gram algorithm (3). Finally, each word is represented by the sum of the embedded sub-words $z_{s}$.


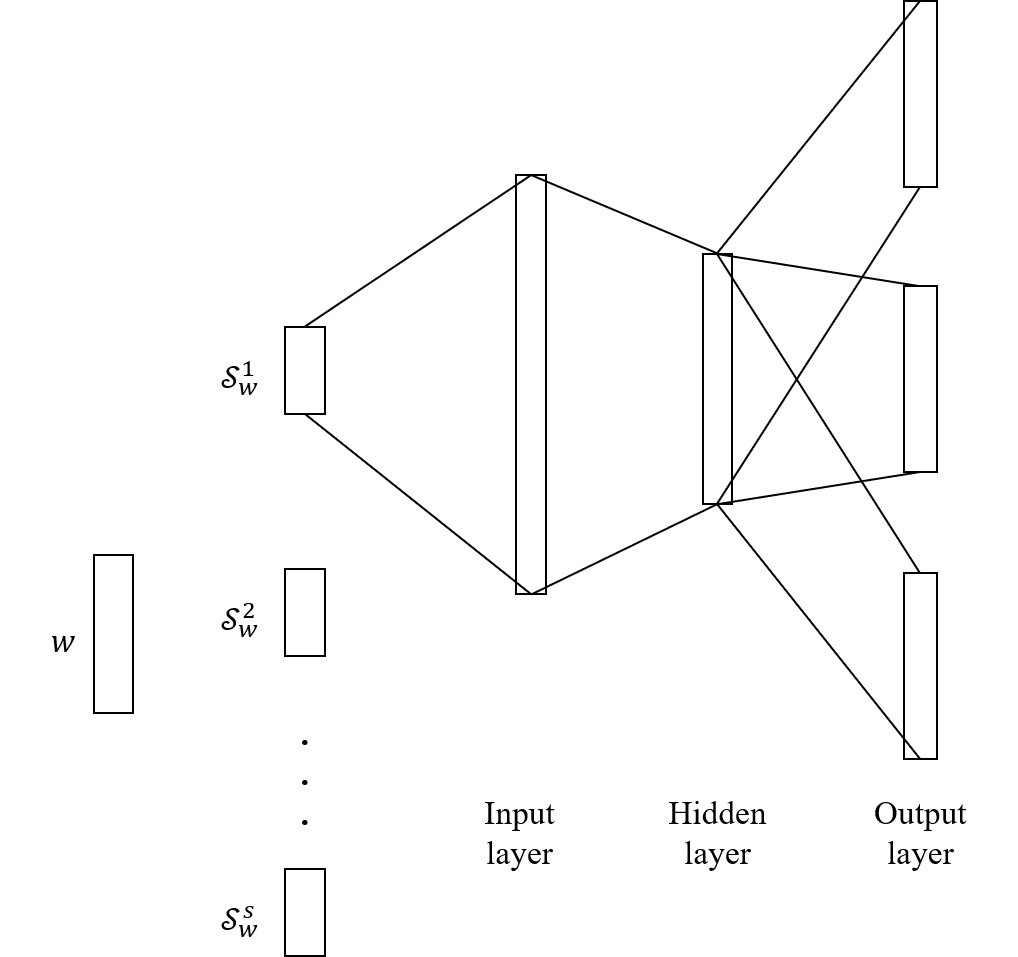
Appendix Figure S3. The architecture of substring embedding model

|  | $s(w, c)=\sum_{s\in\mathcal{S}_{w}} z_{s}U$ | (1) |
| --- | --- | --- |
|  |  |  |

## Structure of our Deep-learning based classification model

### Bi-LSTM Layer

We pass the output of the word representation layer to the Bi-LSTM layer. In this layer, a new representation for each word is created by contextual information from forward and backward directions in a record. The Bi-LSTM is a combination of forward LSTM and backward LSTM. The forward layer memorizes the historical information of the sequence. The backward layer captures the future information of the sequence (4). We concatenate the forward and backward hidden state to obtain hidden state representation for each word. This process helps obtain information of the whole record around every word $W=\left\{ w_{1},w_{2},\cdots,w_{N} \right\}$. We denote all the hidden state of the words as $H=\left\{ h_{1},h_{2},\cdots,h_{N} \right\}$.

### Attention Layer

In a given record, a specific part of the word plays an important role in classifying the pneumonia related dataset. Hence, each word is given appropriate importance in representing the overall semantics of the record (5). The attention layer multiplies the whole hidden states $H$ with the output which is then passed to Bi-LSTM layer as shown below:

|  | $W_{a}=h_{N}H$ | (2) |
| --- | --- | --- |

where $W_{a}$ is an attention weight and $h_{N}$ is the final state of Bi-LSTM. In general, in the translation task, the attention weight is calculated by every combination of hidden states. As the input of model is well represented as the final state implies contextual information of the entire record, we only multiply the final state with whole hidden states. Also, it can save computational resources. After multiplication, extraction of the attentions of each component is passed to the softmax function, which is computed by the following formula:

|  | $W_{a}^{'}=softmax(W_{a}^{i})=\frac{e^{W_{a}^{i}}}{\sum_{i=1}^{N} e^{W_{a}^{i}}}$ | (3) |
| --- | --- | --- |

where $W_{a}^{'}$ is a normalized importance weight of each word by a record. After the softmax function, calculate which word is attended in sentence as follow

|  | $C_{a}=W_{a}^{'}H$ | (4) |
| --- | --- | --- |

where $C_{a}$ is context which is weighted by attention probability. The calculated context is multiplied with weight for normalization.

|  | $O_{a}=W\cdot C_{a}$ | (5) |
| --- | --- | --- |

where $O_{a}$ is the output of the attention and $W$ is the weight of the attention layer, which follows a normal distribution.

### Softmax Layer

We pass the output of the attention layer to the softmax layer for pneumonia classification as shown below:

|  | $\hat{y_{i}}=softmax\left( O_{a}^{i} \right)=\frac{e^{O_{a}^{i}}}{\sum_{i=1}^{N} e^{O_{a}^{i}}}$ | (6) |
| --- | --- | --- |

The cross-entropy loss is used to train this model, which is given by

|  | $loss=-\sum_{i=1}^{c} y_{i}\log\left( \hat{y}_{i} \right)$ | (7) |
| --- | --- | --- |

where $c$ is the number of classes. $y_{i}$ is *i*^th^ class label and $\hat{y}_{i}$ is predicted probability of the *i*^th^ class label.

### Training hyper-parameters in our proposed model

We employed the 300-dimensional Substring embedding. We embedded one record in a 234-dimensional vector. Furthermore, if the number of words in a record is less than the dimension represented, the remaining dimension is padded with a zero vector.

We experimented with 100, 200, 300, 400, and 500-dimensional Doc2Vec embeddings, and found that the 300-dimensional Doc2Vec embeddings achieve the best performance. We set the following parameters for CNN model: convolutional kernel size: 3, number of convolutional filters: 64, dimension of hidden layer in the fully connected layer: 32, dropout keep probability: 0.2, batch size: 32, learning rate: 0.00001.

We use softmax cross entropy loss and Adam optimizer in both CNN and LSTM for the stabilizing the learning. We set the following parameters for LSTM model: number of hidden layers: 150 when using Word2Vec embedding and 200 when using Substring embedding and learning rate: 0.00005.

### Other models compared to our proposed model

- Logistic Regression: The logistic regression is a generalized linear model that concerns the binary classification model, where the link function is the logit or logistic function (6).
- SVM: The SVM is used to construct a hyper-plane or many hyper planes in a high dimensional space, which can then be used for classification (7).
- Naïve Bayes Regression: The Naïve Bayes model deal with the classification problem based on a conditional probability called Bayes rule (8).
- kNN: The kNN calculates the Euclidian distance between new embedding vectors and the training embedding vectors, then assigns the label to new input if there is a minimal Euclidian distance between the new and training embedding vectors (9).
- Decision Tree: The decision tree operates in the form of a tree structure to model the classification. In general, this model assigns the label to new input based on the information gain ratio that is evaluated by entropy (10).
- Random Forest: The random forest construct is a combination of decision trees. This model reduces the error in classification tasks to use of bagging or bootstrap aggregation (11).
- CNN: The CNN is one of the most famous deep learning models that use the convolution layer to extract the local feature. In our work, we use 1D CNN with Doc2Vec embedding, because 1D CNN is mainly used for sequence data (12). Furthermore, Doc2Vec embedding is inspired by the word embedding techniques, it implements the method of distributed representation of context and extracts surrounding word vectors that are specific to a document, in our case a record (13). Therefore, both these methods extract a feature model which is used to compare the performances.

## Appendix Table S1. Comparison of traditional and deep learning models

| Models | Accuracy (%, number) |
| --- | --- |
| **Traditional models** |  |
| Logistic Regression | 83.03% (905/1090) |
| Support vector machine | 73.03% (796/1090) |
| Naïve-Bayes | 64.03% (698/1090) |
| kNN | 75.96% (828/1090) |
| Decision Tree | 71.01% (774/1090) |
| **Deep learning models** |  |
| Doc2Vec+CNN | 73.03% (796/1090) |
| FastText | 90.00% (981/1090) |
| Word2Vec+Bi-LSTM-Attention | 88.99% (970/1090) |
| Proposed Model  (Kor2EngTransfer+Bi-LSTM-Attention) | 91.01% (992/1090) |

kNN = k- Nearest Neighbor, Doc2Vec = Documents to vector machine, CNN = Convolutional neural network

## Appendix Figure S4. Overall processing of our proposed model


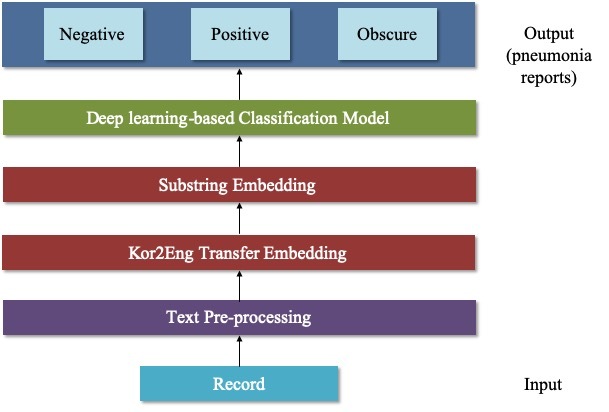


## Appendix Figure S5. AUROC and AUPRC of our proposed model in extra-validation set.


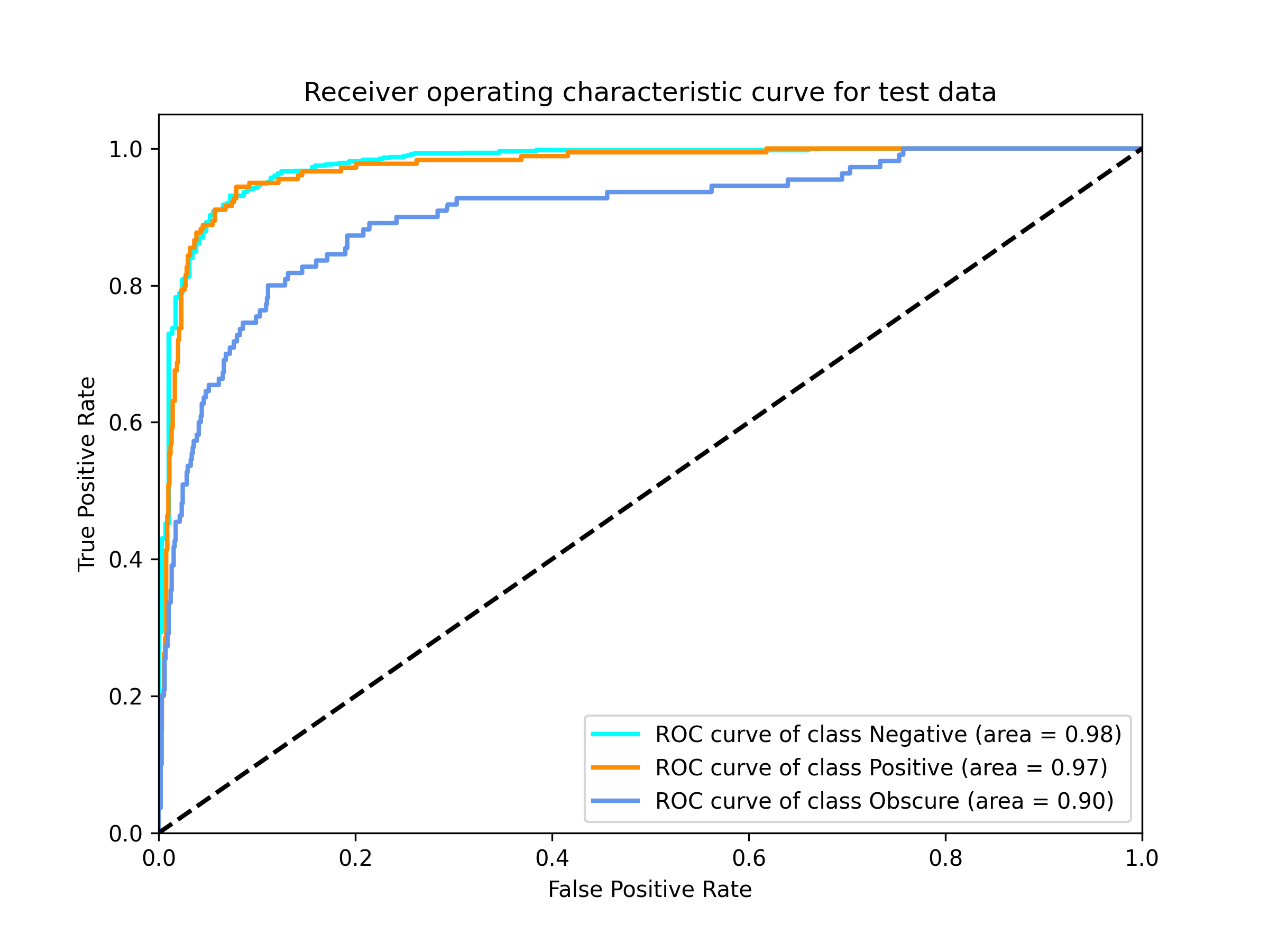

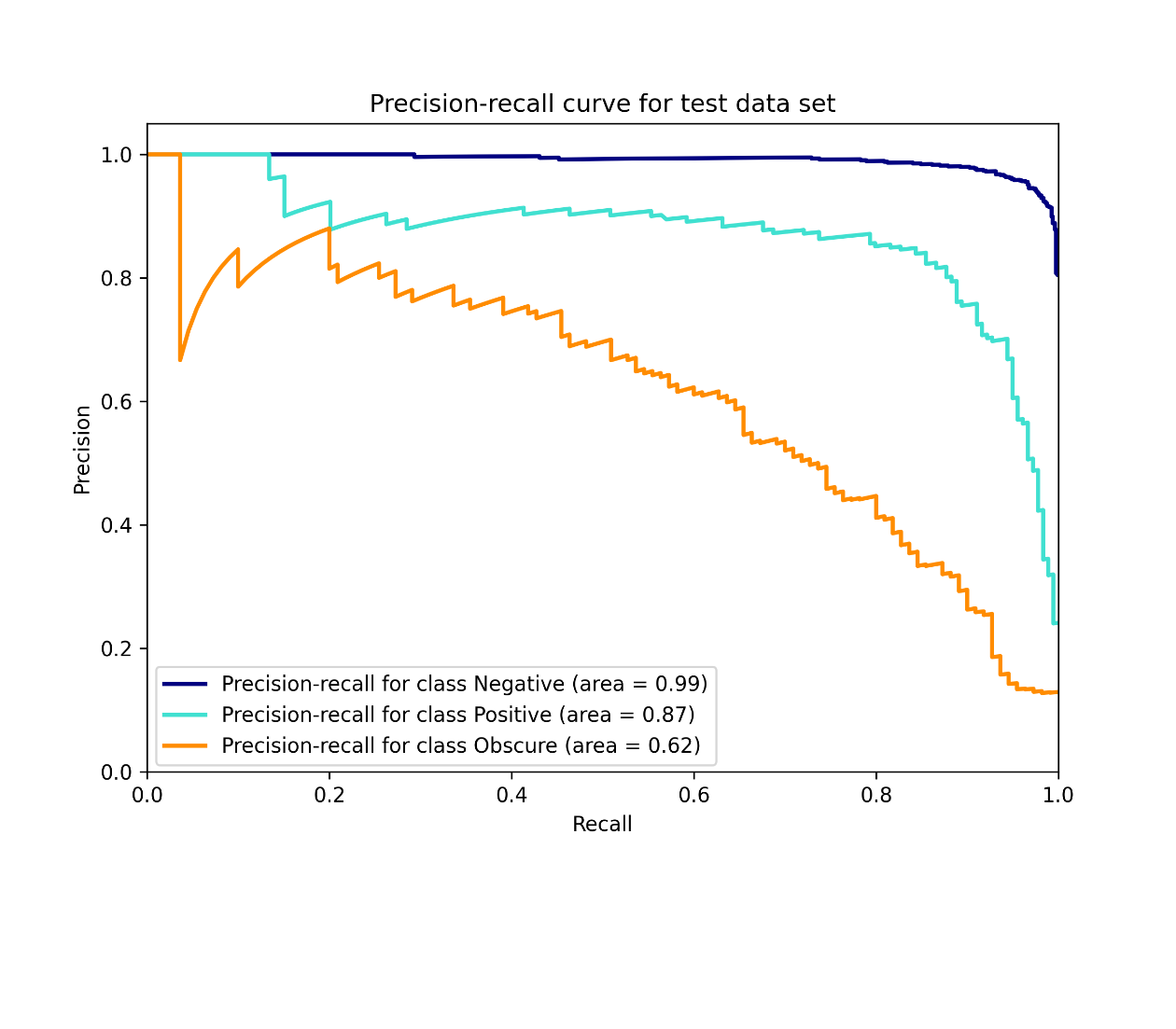


## Reference

1. Wang S, Manning CD. Baselines and bigrams: Simple, good sentiment and topic classification. *50th Annu Meet Assoc Comput Linguist ACL 2012 - Proc Conf* 2012;2:90–94.

2. Lample G, Conneau A, Denoyer L, Ranzato M. Unsupervised machine translation using monolingual corpora only. *6th Int Conf Learn Represent ICLR 2018 - Conf Track Proc* 2018;1–14.

3. Mikolov T, Sutuskever I, Chen K, Corrado G, Dean J. Distributed Representations of Words and Phrases and their Compositionality. *Adv Neural Inf Process Syst* 2013;1389–1399.doi:10.18653/v1/d16-1146.

4. Hochreiter S, Schmidhuber J. Long Short-term Memory. *Neural Comput* 1997;9:1735–1780.

5. Bahdanau D, Cho KH, Bengio Y. Neural machine translation by jointly learning to align and translate. *3rd Int Conf Learn Represent ICLR 2015 - Conf Track Proc* 2015;1–15.

6. Cox DR. The Regression Analysis of Binary Sequences. *J R Stat Soc Ser B* 1958;20:215–232.

7. Cortes C, Vapnik V. Support-Vector Networks. *Mach Learn* 1995;20:273–297.

8. SEBASTIANI F. Machine Learning in Automated Text Categorization. *ACM Comput Surv* 2002;34:1–47.

9. Soucy P, Mineau GW. A simple KNN algorithm for text categorization. *Proc - IEEE Int Conf Data Mining, ICDM* 2001;647–648.doi:10.1109/icdm.2001.989592.

10. Quinlan JR. Induction of decision trees. *Mach Learn* 1986;1:81–106.

11. Breiman L. Random_Forest. *Mach Learn* 2001;45:5–32.

12. LECUN Y, LEON B, Yoshua B, Patrick H. Gradient-Based Learning Applied to Document Recognition. *Biochem Biophys Res Commun* 1998;86:2278–2324.

13. Djuric N, Zhou J, Morris R, Grbovic M, Radosavljevic V, Bhamidipati N. Distributed Representations of sentences and documents. *Proc 24th Int Conf World Wide Web - WWW ’15 Companion* 2014;32:29–30.
